# Structural mass spectrometry decodes domain interaction and dynamics of the full-length Human Histone Deacetylase 2

**DOI:** 10.1016/j.bbapap.2022.140759

**Published:** 2022-03-01

**Authors:** Zoja Soloviev, Joshua M.A. Bullock, Juliette M.B. James, Andrea C. Sauerwein, Joanne E. Nettleship, Raymond J. Owens, D. Flemming Hansen, Maya Topf, Konstantinos Thalassinos

**Affiliations:** a Institute of Structural and Molecular Biology, Division of Biosciences, University College London, London WC1E 6AR, UK; b Institute of Structural and Molecular Biology, Birkbeck College, University of London, London WC1E 7HX, UK; c PPUK, Research Complex at Harwell, Rutherford Appleton Laboratory, Oxford OX11 0FA, UK; d Division of Structural Biology, University of Oxford, The Wellcome Centre for Human Genetics, Headington, Oxford, UK; e Centre for Structural Systems Biology, Heinrich-Pette-Institut, Leibniz-Institut für Experimentelle Virologie, Hamburg, Germany

**Keywords:** HDAC2, Human Histone Deacetylase 2, HDACs, Human Histone Deacetylases, MS, Mass Spectrometry, IM-MS, Ion Mobility Mass Spectrometry, PTMs, Post-Translational Modifications, Ion Mobility Mass Spectrometry (IM-MS), Crosslinking, Molecular modelling, Intrinsically-disordered proteins, Human Histone Deacetylase 2 (HDAC2)

## Abstract

Human Histone Deacetylase 2 (HDAC2) belongs to a conserved enzyme superfamily that regulates deacetylation inside cells. HDAC2 is a drug target as it is known to be upregulated in cancers and neurodegenerative disorders. It consists of globular deacetylase and C-terminus intrinsically-disordered domains [1–3]. To date, there is no full-length structure of HDAC2 available due to the high intrinsic flexibility of its C-terminal domain. The intrinsically-disordered domain, however, is known to be important for the enzymatic function of HDAC2 [1, 4].

Here we combine several structural Mass Spectrometry (MS) methodologies such as denaturing, native, ion mobility and chemical crosslinking, alongside biochemical assays and molecular modelling to study the structure and dynamics of the full-length HDAC2 for the first time. We show that MS can easily dissect heterogeneity inherent within the protein sample and at the same time probe the structural arrangement of the different conformers present.

Activity assays combined with data from MS and molecular modelling suggest how the structural dynamics of the C-terminal domain, and its interactions with the catalytic domain, regulate the activity of this enzyme.

## Highlights

• Human Histone Deacetylase 2 shows increased enzymatic activity in truncated form.
• Structural mass spectrometry allows to understand protein architecture with intrinsically disordered and globular regions.
• Molecular modelling integrates mass spectrometry data and provides insight into enzymatic activity.
• Entry to the Human Histone Deacetylase 2 active site could be occluded by its intrinsically-disordered domain.

## Graphical abstract

**Figure:**
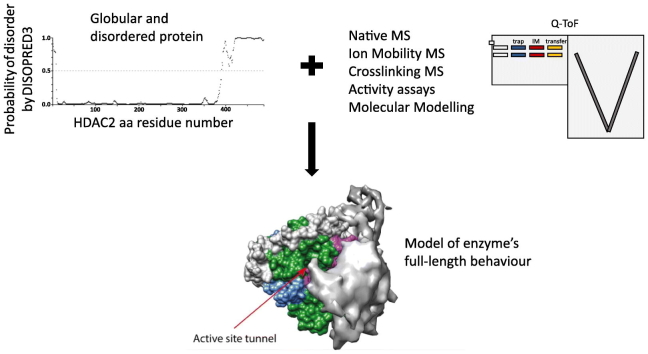

## 1 Introduction

One mechanism of regulating gene expression during the different developmental stages of a cell, is the epigenetic modification of the chromatin structure, specifically the histone tails. Histone proteins form the core of nucleosomes, which are structural units of chromatin responsible for compacting genomic DNA [5]. Post-translational histone tail modifications are achieved through the activity of transcriptional regulators that recognise, bind to a particular DNA sequence, and either add or remove Post-Translational Modifications (PTMs) [6]. One of the most common PTMs is lysine acetylation, whereby acetyl groups are added to the amino acid side-chains, resulting in neutralisation of the positive charge [7]. Acetylation and deacetylation are regulated by two groups of enzymes: Histone Acetyl Transferases (HATs) and HDACs. HATs catalyse acetylation of histone tails, which results in the relaxation of the chromatin structure and a subsequent increase in the expression of the underlying genes. Conversely, HDACs remove acetyl groups, which results in a collapse of the chromatin structure and down-regulation of gene expression. HDACs and other acetyl modifiers do not only target histone tails, but also other proteins inside the cell, such as p53 and other transcription factors [[7], [8], [9]]. High-throughput proteomics studies, have identified over 3000 different acetylation sites targeted by HDACs [10].

There are four classes of human HDACs, all catalysing the removal of acetyl groups from lysine side-chains, with zinc-dependent HDACs present within three of these classes (I, II and IV).

HDAC1 and HDAC2 are among the first two discovered mammalian HDACs [11,12]. They share over 84% sequence identity [13], but this sequence identity is unevenly distributed between them. The catalytic globular domains share over 90% identity, while the flexible C-terminal regions are 72% identical and have different PTM patterns [1].

Most human HDACs form an enzymatic core of multi-protein complexes. HDAC2 is known to be a catalytic part of complexes such as Sin3, Nucleosome Remodelling Deacetylase (NuRD), Co-Repressor for Element-1 Silencing Transcription factor (CoREST) and more recently identified Mitotic Deacetylase Complex (MiDAC) [14,15]. These complexes are often called co-repressor complexes since they bind to the transcription factors inside the cell and alter gene expression [16,17]. Recombinantly expressed HDAC2 has been shown to exhibit catalytic activity even outside the co-repressor complex [18]. This suggests that the deacetylase functionality of the co-repressor complexes relies on HDAC2 and that the remaining proteins in the complex predominantly function as chromatin binding, re-modelling proteins, as well as regulators of activity [16].

The catalytic site of HDAC2 contains a zinc ion, coordinated by two aspartic acids in positions 181 and 269 and a histidine in position 183. HDAC2 has three different regions: oligomerisation, activity and regulatory. There are partial crystal structures available for HDAC2 (PDB IDs: 3MAX, 4LXZ, 4LY1, 5IX0, 5IWG, 6G3O, 6WBZ, 6WBW, 6XEB, 6XDM and 6XEC), but they all lack the last 100+ residues on the C-terminus, as these residues cannot be crystallised because of intrinsic flexibility as predicted by DISOPRED3 [3]. Importantly, the flexible C-terminus is responsible for regulating the activity of the enzyme as shown by domain-swapping experiments. These experiments showed that by attaching the C-terminal region of HDAC1 to HDAC2 protein and vice versa, the activity profile between these two enzymes was swapped as the PTM profile of the C-terminal regulatory domains was mirrored [4]. There are currently four FDA-approved inhibitors available [[19], [20], [21]], but they are non-specific, targeting all 11 HDAC enzymes and giving rise to off-target side-effects. The understanding of the unique disordered C-terminus in HDAC2 that regulates enzymatic activity, could provide more clarity about the desired Molecular Mechanism of Action (MMoA) for future drugs. Additionally, better understanding of the full-length enzyme could guide dosage prediction required for HDAC2 inhibitors to work efficiently in humans. Understanding how the C-terminal domain interacts with the core globular body will not only reveal the role of the C-terminal domain in regulating HDAC2 function but would also help to understand its role in co-repressor complex formation.

In recent years, MS has been widely used in structural biology to answer a wide variety of questions, ranging from identification of the primary protein sequence and PTMs, to epitope mapping and protein complex assembly determination [[22], [23], [24]]. It serves as a complementary technique to conventional structural biology methods such as Nuclear Magnetic Resonance (NMR), Electron Microscopy (EM) and X-ray crystallography. A unique advantage of MS is that it can overcome some of the size and flexibility limitations imposed by other techniques thanks to its ability to utilise low sample concentrations, its speed of analysis, and ability to analyse heterogeneous and polydisperse samples.

Even though at its core MS measures the mass-to-charge ratio of ionised molecules there are a number of different experimental workflows that provide complementary information regarding a protein or protein complex. Native MS allows the study of folded proteins and protein complexes while preserving non-covalent interactions [25]. Coupling Ion Mobility to MS (IM-MS) adds an additional dimension of separation that allows one to interrogate the conformation and conformational flexibility of proteins [26,27]. The IM-MS experiment reports on the time it takes an ion to traverse a mobility cell filled with inert gas, which is related to the ion's shape, charge and mass [28]. The resulting Arrival Time Distributions (ATDs) can be converted to Collision Cross Section (CCS) and compared to existing crystal or NMR structures as well as homology models, and in many cases shown to be in excellent agreement [[29], [30], [31]]. Native and IM-MS have advanced a lot in the study of soluble as well as membrane proteins and are not limited by dynamics, heterogeneity or size of the protein system [[32], [33], [34], [35]]. Crosslinking MS can provide information on protein-protein, or protein inter- and intra-domain interactions by studying covalently-linked peptides using MS. Covalent protein crosslinking is performed in-solution prior to sample digestion with proteases into peptides, and hence these studies provide additional information on protein fold and solvent accessibility; these data could be especially valuable to help understand behaviour of the intrinsically-disordered proteins that are challenging to study using high-resolution technique [36,37].

Here we describe how a combination of different structural MS techniques, activity assays and molecular modelling reveal an ensemble of structural models describing the full length HDAC2 protein. These data allowed us to propose a mechanism of action of the C-terminal regulatory domain. We showcase how the complementary information from native MS, IM-MS, crosslinking MS and integrative structure modelling could be used together to create a fuller understanding of the biological system.

## 2 Materials and methods

Chemical and enzyme reagents if not otherwise stated, were purchased from Sigma-Aldrich. All LC-MS/MS analyses were performed using LC-MS grade solvents and water from Sigma-Aldrich.

### 2.1 Protein expression and purification

Full-length HDAC2 construct FL-HDAC2-FLAG-8xHIS (Supplementary, Table S1) was cloned into pOPINE and a baculovirus constructed for protein production in insect cells (PMID: 17317681) *Sf9* cells (2.5 L) were infected with the amplified virus at an MOI of approximately 100:1 and after 72 h, cells were harvested by centrifugation at 5000 x*g* for 30 min. Pellets were frozen and transferred to UCL for two-step purification using affinity (NiNTA) chromatography followed by the preparative Size Exclusion Chromatography (SEC).

C-terminal construct - 6xHIS-TEV-HDAC2(338-388aa) was synthesised by GenScript in pET-29b + plasmid. Protein was expressed in *E.coli* BL21(DH3) cells (Novagen) grown in 1 L LB. T7 promoter-driven expression was induced with 1 mM Isopropyl β- d-1-thiogalactopyranoside (IPTG) and cells were left in the shaker (220 rpm) at 20 °C overnight. Cells were harvested and pellets frozen at −80 °C until purification. Three step purification was performed using affinity (NiNTA) chromatography, Tobacco Etch Virus (TEV) protease cleavage followed by a second step of affinity chromatography and preparative (SEC).

For both purification protocols, cell pellets were re-suspended in lysis buffer (25 mM Tris-HCl, pH 8, 300 mM NaCl, 5% glycerol, 2 mM β-mercaptoethanol, and 5 mM CaCl_2_). Volume of the lysis buffer was x5 times weight of the pellet. Lysis buffer was supplemented with Benzonase (Novagen) and EDTA-free protease inhibitor cocktail (Roche). Dissolved pellets were put through the French Press Cell (stored at 4 °C) at 1250 psi. Lysate was spun at 40,000 rpm (45 Ti rotor) for 30 min, followed by filtration using 0.45- μm and 0.22-μm filter. Filtered supernatant was loaded onto pre-equilibrated NiNTA column (HisTrap, 5 mL, GE Healthcare) at a rate 1 mL/min using peristaltic pump. Column was washed with the lysis buffer and wash buffer (25 mM Tris-HCl, pH 8, 300 mM NaCl, 5% glycerol, 2 mM β-mercaptoethanol, 5 mM CaCl_2_ and 5 mM imidazole). The bound protein was eluted from a column on ÄKTA purifier with a linear imidazole gradient (5–500 mM). Collected fractions were assessed on SDS-PAGE, and fractions containing protein were pooled, concentrated, and purified on preparative SEC (S200 (FL)\S75 (truncated), GE Healthcare) on ÄKTA purifier using gel filtration buffer (25 mM Tris-HCl pH 8.0, 150 mM NaCl, 5 mM CaCl_2_, 5 mM DTT and 5% glycerol). For C-terminal construct additional step was performed prior to SEC, sample was buffer exchanged into SEC buffer (to remove Imidazole), TEV protease (His-tagged) cleavage was performed using 1:10 TEV protease:HDAC2 construct ratio. Efficiency of tag removal was monitored on SDS-PAGE. Once tag removal was complete, sample was put thought NiNTA column and supernatant containing HDAC2 C-terminal construct was collected, concentrated and subjected to the SEC (S75, GE Healthcare). Collected fractions were analysed on SDS-PAGE, fractions containing protein were pooled. Samples were flash frozen in liquid N_2_ and stored at −80 °C until analysis.

### 2.2 Protein gels

SDS-PAGE was run using pre-cast gels (Tris-Glycine, 4–20% from Novagen) at 200 V in MES buffer for 50 min. Gels were stained using InstantBLUE (Expedion) and de-stained in water.

### 2.3 Native and ion mobility mass spectrometry

Sample from stock buffer was buffer exchanged into 300 mM ammonium acetate using BioSpin 6 (BioRad) spin columns or centrifugal filters Amicon Ultra 0.5 mL 10MWCO (HDAC2 C-terminal construct) and 30 MWCO (HDAC2 FL) (both from Millipore). After buffer exchange concentration of the protein was adjusted to 15 μM using Qubit fluorometer (Invitrogen). Native MS and IM-MS experiments were carried out on the hybrid Synapt Quadrupole Time-of-Flight (Q-ToF) instrument (Waters) described previously [28]. Gentle instrumental conditions were used to preserve protein conformation: Capillary voltage 1.0–1.2 kV, cone voltage 30–40 V, trap/transfer energies – 6/4 V. For IM-MS acquisition the source pressure was 4.0 mbar, IMS cell pressure 5.2 × 10^−1^ mbar and trap cell pressure 2.3 × 10^−2^ mbar. IMS travelling wave and velocity were adjusted to achieve optimal separation. Native MS data were analysed using MassLynx (4.1, Waters), and IM-MS data were analysed using Driftscope (2.8, Waters) and *in-house* software Amphitrite [26]. Experimental CCS values were extracted from arrival time distributions using a set of standard proteins (β-lactoglobulin, Concanavalin A and Bovine serum albumin, all from Sigma) as previously described [38].

### 2.4 Intact mass unfolding mass spectrometry

Protein unfolding was performed using ZipTips (C4, Milipore). Samples were buffer exchanged into 70% acetonitrile containing 0.1% trifluoroacetic acid. All samples were delivered into mass spectrometer (Synapt G2Si Q-ToF, Waters) using *in-house* prepared gold-coated capillaries (1.00 mm × 0.78 mm, Harvard apparatus), pulled using a needle-puller (P-97, Sutter Instrument) and coated with gold using a sputter-coater (SC7620, Emitech) as described previously [39].

### 2.5 Limited proteolysis

Limited proteolysis experiments were performed using sequencing grade trypsin (Promega). The experiments were performed by adding 1:10,000 M ratio of trypsin:HDAC2 and incubating at room temperature for 3 h. Following incubation, samples were buffer exchanged as described in section 2.3 and used to acquire native mass spectra, or in activity assays (section 2.7).

### 2.6 Crosslinking and LC-MS/MS experiments

For LC-MS/MS, a sample of interest was run on a SDS-PAGE and the band of interest was excised, cut into small pieces followed by a series of steps to de-stain, reduce and alkylate protein (shrink/swell in acetonitrile/50 mM ammonium bicarbonate respectively; reduction in 10 mM dithiothretol for 30 min and alkylation in 55 mM iodoacetamide for 30 min). Following that 90 μL of 10 ng/μL trypsin (Promega, sequencing grade) was added to the gel band and digested at 37 °C o/n. Samples were then aspirated, transferred to clean tubes, dried and re-solubilised in 0.1% formic acid in water prior to analysis.

For crosslinking experiments, samples were buffer exchanged from storage buffer into 20 mM HEPES. D0/D12 Bis-(Sulfosuccinimidyl) Suberate (BS3) and Di-Succinimidyl Suberate (DSS) crosslinkers were used (both from Creative molecules). Crosslinkers were added to protein sample at a final molar ratio of 1:100 protein:crosslinker. Reactions were incubated for 1 h at RT and quenched with a final concentration of 50 mM ammonium bicarbonate. Samples were run on SDS-PAGE and digested as described above. Digested samples were dried and dissolved in 20 μL SEC buffer (30% acetonitrile and 0.1% TFA), samples were spun down using bench top centrifuge for 10 min at maximum speed, and 15 μL of sample was loaded onto pre-equilibrated ÄKTAmicro FPLC system using SEC buffer and Superdex PC3.2/3.0 column (GE Healthcare). Fractions were collected at 50 μL/min flow rate every two minutes. Individual fractions were aspirated, transferred to clean tubes, dried and re-solubilised in 0.1% formic acid in water prior to analysis. LC-MS/MS analyses were performed using nanoACQUITY UPLC system connected to Synapt G2Si Q-ToF (both Waters) and crosslinks were analysed as previously discussed [40].

### 2.7 Fluorescence activity assays

The enzyme substrate Boc-Lys(AC)-7-amino-4-methylcoumarin (MAL) was synthesised as previously described [41] and stored at 2 mM in DMSO until use. All assays were performed in 96-well plates. For each condition, HDAC samples were prepared in assay buffer (50 mM Tris-HCl, pH 8.0, 137 mM NaCl, 2.7 mM KCl and 1 mM MgCl_2_), and dispensed at 10–80 ng/well. Additional wells were used for ‘no enzyme’, ‘no developer’ and limited proteolysis controls (trypsin only). The HDAC samples were mixed with 25 μL of MAL substrate diluted in assay buffer from stock concentration to 80 μM. Samples were mixed by pipetting solution up-and-down and incubated at room temperature for 30 min. After incubation, 50 μL developer solution (10 mg/mL trypsin and 2 μM Trichostatin A inhibitor (Enzo Life Sciences)) was added to each well and left for 15 min incubation before measuring the fluorescence signal. The plate was imaged using a BMG FLUOstar Optima plate reader at 380 nm excitation and 460 nm emission. Gain was adjusted to 95% based on the well containing most enzyme. The reading was taken 3 times and results averaged for each well. All assays were performed in triplicates.

### 2.8 Molecular modelling

Molecular modelling using experimental crosslinking restraints was performed using the Integrated Modelling Platform (IMP) [42]. Each crosslink was implemented as part of the Bayesian inferential approach that determines the probability that the model comes from the data given the experimental data and other prior knowledge. Monte Carlo sampling was used to sample the Bayesian probability space, generating a total of 150,000 models from 20 separate modelling runs.

At first, coarse-grain modelling was performed where 4 amino acids were represented as one bead followed by finer-grain modelling with each amino acid represented by a single bead. For both modelling experiments, the top 100 models based on IMPs probability score were clustered using k-means clustering based on the RMSD of the centroids of the beads. This score includes the experimental crosslinking data. Experimental crosslinks were then used again with the Matched and Non-accessible Crosslink Score (MNXL) to select the top 10 models [[43], [44], [45]]. Theoretical CCS values of the models were calculated using IMPACT [29].

### 2.9 Disorder prediction

Domain structure and disorder prediction for HDAC2 was calculated using PSIPRED and DISOPRED3 [2,3], where C-terminal domain is predicted to be disordered (Fig. S2).

## 3 Results

### 3.1 Intact mass spectrometry studies indicate structural flexibility

**Fig. 1:**
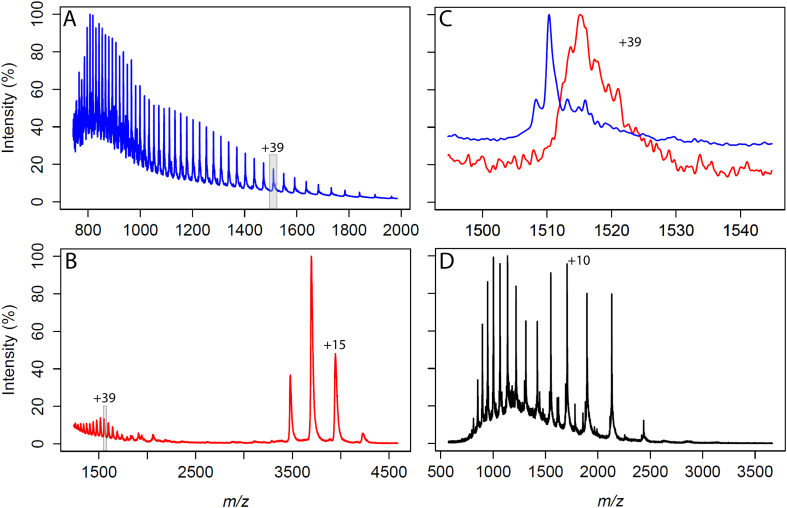
Mass spectra of different HDAC2 constructs. (A) Mass spectrum of the full-length HDAC2 under unfolding conditions (0.1% TFA, 70% acetonitrile and 29.9% water), where the charge state +39 is highlighted in grey. (B) Mass spectrum of the full-length HDAC2 under native conditions (300 mM ammonium acetate), where two different charge state envelopes can be seen. In grey is the highlighted charge state +39. (C) Overlay of charge state +39 between the unfolding (blue) and native spectra (red). The mass difference between the two peak tops suggests the non-covalently bound calcium and zinc ions are present under the native conditions, but absent under the unfolding conditions. (D) Mass spectrum of HDAC2 C-terminus in isolation under native conditions (300 mM ammonium acetate), exhibiting an unfolded-like charge state distribution. (For interpretation of the references to colour in this figure legend, the reader is referred to the web version of this article.)

Native and unfolding MS was performed on the full-length HDAC2 protein (Fig. 1A-C) expressed in insect cells (*Sf9*). The C-terminal domain expressed in isolation in bacterial (*E.coli*) cells was also analysed on native MS (Fig. 1D). Native MS of the full-length protein shows two separate charge state distributions (Fig. 1B) that are well separated in the spectrum (high charge state distribution annotated with CS +39 and low charge state distribution annotated with CS + 15).

In nESI, the protein is protonated at different basic residues on the surface, which results in multiple peaks, where peaks correspond to different charged states. A low-charge state distribution, centered around charge state +16 (Fig. 1B) is likely to correspond to a more folded-like species as fewer residues are exposed for protonation, while the high-charge state distribution centered around charge state +40 is likely to represent a more unfolded-like protein. However, it is known that the HDAC2 C-terminus is highly flexible and contains a high number of charged amino acid residues such as 20 lysines (out of 41 lysines in the full protein sequence), 6 arginines and 3 histidines. It was hypothesised that such a strong accumulation of charged residues and the intrinsic flexibility of the C-terminus was contributing to two different conformations that resulted in two charge state distributions. It is possible that in an open state the C-terminus would have increased the number of protonation sites available for ionisation, while a closed conformation of the C-terminus would give rise to the lower charge state species.

Experiments were performed to compare HDAC2 protein under native and unfolding conditions (Fig. 1A-C). It is known from the literature that HDAC2 is a metal-binding protein and currently-available crystal structures contain zinc and calcium ions, which were also used in expression media or purification buffers in the experiments described here. Such non-covalent protein-metal interactions are usually preserved in native MS [46] experiments, but would be lost when the protein is unfolded. Interestingly, the high charge state species in the native mass spectrum of HDAC2 show a mass shift when compared to the same charge state from the unfolded spectrum (Fig. 1A). The mass shift corresponds to the mass of the metal ions (zinc – 65 Da, and calcium – 40 Da) still present in the native mass spectrum but lost in the unfolding experiment. These data suggest that the fold of the globular domain of HDAC2 (containing metal ions) is preserved under the native MS conditions across two different charge state distributions. It is likely that the two charge state distributions are due to the different configuration of the C-terminal domain. In addition, the native mass spectrum was acquired for a C-terminal domain expressed in isolation in bacterial cells (last alpha helix and flexible region only, Fig. 1D). This mass spectrum shows the protein to be in an unfolded-like state even though it was analysed under native conditions, which supports the hypothesis that the C-terminal region results in two different charge state distributions (Fig. 1B).

### 3.2 Truncated protein changes appearance of two different charge-state distributions

**Fig. 2:**
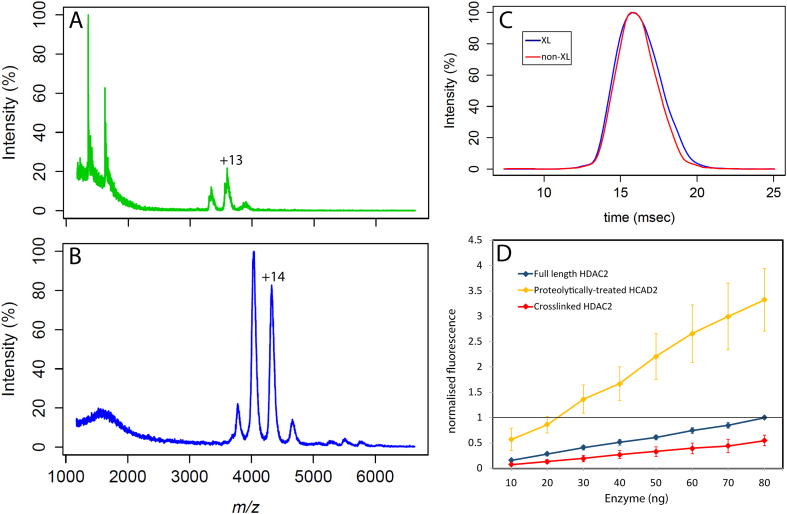
MS and enzymatic activity assay results of the modified HDAC2. (A) Mass spectrum of proteolytically-treated HDAC2 (with trypsin) under native conditions (300 mM ammonium acetate). One charge state envelope can be observed between 3000 and 4000 *m/z*. The two peaks between 1000 and 2000 *m/z* are charge states +6 and + 5 and are predicted to correspond to a peptide sequence from the C-terminus of HDAC2 that results from the limited proteolysis treatment with trypsin (Supplementary Table S2). (B) Native mass spectrum of the full-length HDAC2 crosslinked with the BS3 reagent. A folded-like charge state envelope of HDAC2 protein can be seen between 3500 and 5000 *m/z*. (C) Overlay of the IM-MS output for charge state +14 from crosslinked (blue) and non-crosslinked sample (red) of the full-length HDAC2. (D) Fluorescent enzymatic activity assay results of three different samples: non-crosslinked HDAC2 (blue), proteolytically-treated HDAC2 (yellow) and crosslinked HDAC2 (red), which show different activity readouts. Assays were repeated 3 times and error bars standard deviation between repeats. (For interpretation of the references to colour in this figure legend, the reader is referred to the web version of this article.)

To test whether the two distinct charge state distributions (Fig. 1B) were due to the C-terminus, we performed limited proteolysis and crosslinking experiments. After the limited proteolysis, Native MS was used to analyse the resulting protein (Fig. 2A). The spectrum shows a low charge state distribution centered around CS +13 and two peaks at low *m/z* values corresponding to a C-terminal fragment (see Supplementary Tables S2 and S3 for mases). Peptides corresponding to the C-terminal fragment were identified by LC-MS/MS from the full-length HDAC2 sample, but not from HDAC2 previously subjected to limited proteolysis (Supplementary Fig. S1). There were also peptides missing on the N-terminus fragment, but experimental masses were not matched corresponding to these fragments (Supplementary Fig. S1).

### 3.3 Ion mobility suggests no structural re-arrangement upon crosslinking

Interestingly, upon covalent crosslinking of the sample, the native mass spectrum (Fig. 2B) showed decrease in high charge state species at low *m/z* (Figs. 1A and 2B). The isolated CS +14 from non-crosslinked and crosslinked samples of the full-length HDAC2 showed very similar arrival time distributions (Fig. 2C), indicating that there are no significant structural effects introduced by the crosslinking. The reduction in the high charge state species in the native mass spectrum of the crosslinked sample (Fig. 2B) implies that during the crosslinking reaction in solution, there might be a fast exchange between open and closed states of the C-terminus. As the C-terminus comes close to the globular body, the crosslinking reagent with the linker constraint of 33 Ångstroms (Cα-Cα between 2 lysines) can capture that state and link it to the globular body [43].

### 3.4 Truncated HDAC2 has upregulated deacetylase activity

To check the activity of the three different HDAC2 conditioned samples we performed activity fluorescent assays on: non-treated, proteolytically treated with trypsin to cleave the C-terminus and crosslinked HDAC2 samples. Interestingly, the proteolytically-treated samples showed upregulated deacetylase activity when compared to the non-treated sample, while the crosslinked sample proved to be less active than the control (Fig. 2D). These data suggest that not only the C-terminal domain is flexible, which is reflected in the native mass spectra (Figs. 1A, 2A and B), but that it is also implicated in regulating the activity of the globular deacetylase domain.

### 3.5 Crosslinking results suggest co-localisation of inter-domain crosslinks

Crosslinking experiments were performed in order to structurally assess the in-solution dynamics of the full-length HDAC2 protein. We looked at a combination of crosslinks between different domains and within the same domain. Of specific interest was the selection of crosslinks between the globular and the intrinsically-disordered C-terminus.

**Fig. 3:**
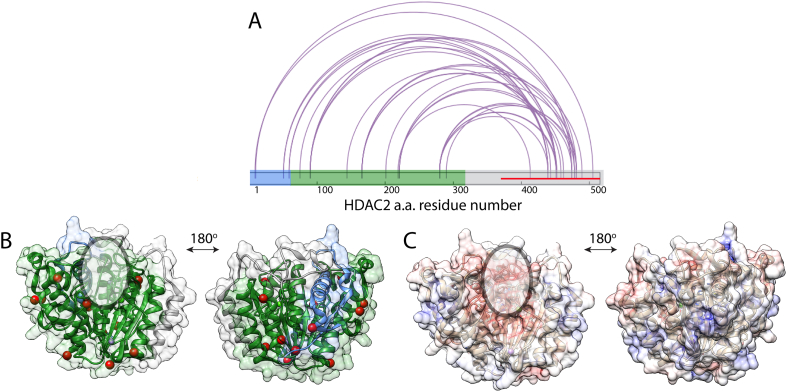
Crosslinking results of full-length HDAC2. (A) Map of crosslinks connecting the globular and intrinsically-disordered C-terminus (red line). HDAC2 sequence is coloured by regions (blue - oligomerisation, green - deacetylase and grey - regulatory regions. Figure was generated using xiNET [47]. (B) Globular domain of the HDAC2 crystal structure (PDB ID: 3MAX) coloured by regions (blue - oligomerisation, green - deacetylase and grey - regulatory regions with all the lysines crosslinked to the disordered region marked with red spheres. (C) Electrostatic map of the HDAC2 globular domain (PDB ID: 3MAX, red – negative potential, white – near neutral and blue – positive potential). Active site channel is indicated by the oval in (B) and (C).(For interpretation of the references to colour in this figure legend, the reader is referred to the web version of this article.)

Crosslinking reagent targeting primary amines was selected because of the known flexibility of the C-terminus and the number of lysine residues (20 lysines out of total 41 are located on the C-terminus). Crosslinks connecting globular and intrinsically-disordered C-terminus were specifically selected from the LC-MS/MS data and further analysed. In total, more than 150 crosslinks between the intrinsically-disordered and globular domains were identified. Out of total the number of crosslinks, the unique high-scoring crosslinks (xQuest score above 20) were selected. The map of the crosslinked residues is shown in Fig. 3A. The lysine residues of the globular domain that were linked to the intrinsically-disordered region were mapped onto the existing crystal structure. It can be seen that these residues localise on two planes of the protein (Fig. 3B). Interestingly, one of the regions is in proximity to the active site of the protein, where metal ions are located as indicated in Fig. 3B and C. The electrostatic map of globular domain (Fig. 3C) shows that positive sites (lysines) of the protein were targeted in crosslinking reactions and the active site region is encapsulated in-between these as a negatively-charged patch.

### 3.6 Molecular modelling suggests clustering of the intrinsically-disordered region in the proximity to the active site on the globular domain

**Fig. 4:**
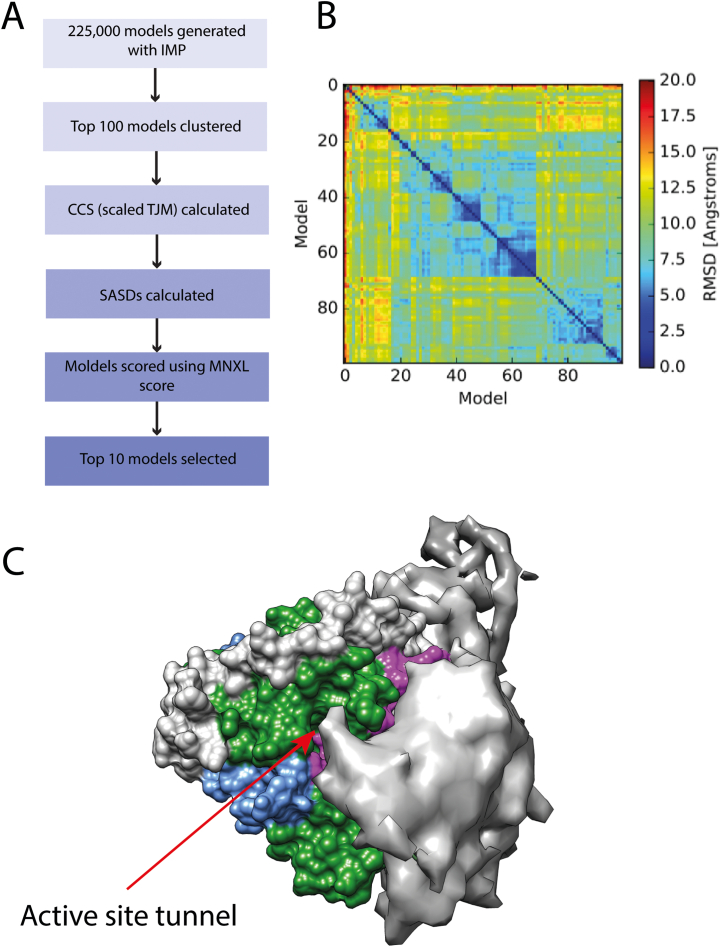
Molecular modelling of the intrinsically-disordered domain of HDAC2. (A) Flow-chart showing the steps employed in the modelling process. IMP and MNXL software were implemented to model and score the resulting models [42,45]. (B) RMSD clustering of the top-100 models. (C) Crystal structure of HDAC2 (PDB ID: 3MAX) coloured by domains (blue - oligomerisation, green - deacetylase and grey - regulatory domains and magenta – flexible loop region) overlaid with the top-10 scoring models of the unstructured region. Active site tunnel is indicated with the arrow. (For interpretation of the references to colour in this figure legend, the reader is referred to the web version of this article.)

Molecular modelling was performed using a combination of crosslinking data calculations and our recently-developed scoring algorithm MNXL (Fig. 4A).

Following the initial coarse-grain modelling, a second modelling step was applied where each amino acid was represented as a single bead. The flowchart (Fig. 4A) shows the approach used to filter our pool of models. The top 100 models were selected from the clustering results (Fig. 4B) and further validated by using the calculated SASDs scored against the crosslink dataset using the MNXL score [43,44]. The remaining top 10 scoring models were selected as a representative dataset and the space they occupy shown in Fig. 4C. Interestingly, this region is close to the active site tunnel of the protein, however, it is not occluding the active site. This is to be expected considering the activity tests shown (Fig. 2D). It is important to note that the activity of the HDAC2 enzyme is increased upon removal of the C-terminus, but the enzyme shows high activity even when tested in its full-length.

## 4 Discussion

### 4.1 Human Histone Deacetylases in development and disease

Human HDAC2 is one of the key nuclear-localised enzymes, its activity is important in development and it has been shown to be a relevant drug target in a number of diseases such as carcinomas and neurodegenerative disorders. However, currently available small molecule inhibitors do not distinguish selectively enough between eleven different HDAC isoforms, leading to side effects. Better structural understanding of the activity control of HDACs could lead to the discovery of more selective inhibitor molecules.

### 4.2 C-terminal domain flexibility

It was shown in the native mass spectra that HDAC2 co-exist as two different conformational species: a native-like conformer, and an unfolded-like conformer, as shown by the low and high charge state distributions respectively (Fig. 1B). Our data suggest that this behaviour of the protein is due to the chimeric nature of the intrinsically-disordered domain. This implies that the unfolding behaviour of the protein is due to the flexible C-terminal region, and the high number of charged residues that are more accessible for protonation during the ionisation process. This was further confirmed by probing C-terminal region expressed in isolation using native MS, which showed the unfolded-like behaviour (Fig. 1D).

### 4.3 Intrinsically-disordered proteins in the gas phase

It is commonly-accepted that intrinsically-disordered proteins could collapse in the gas phase. There are a number of previous studies showing gas-phase collapse of proteins containing regions of high disorder [48,49]. However, it was also shown that the behaviour of the intrinsically-disordered proteins could also depend on the ionisation conditions [50] as well as chemical composition of the protein, and various intrinsically-disordered proteins will behave differently in the gas phase [51]. The experimental CCS values reported here for low charge-state distribution are smaller when compared to the theoretical CCS values estimated from models (Supplementary tables S4 and S5). This suggests that the flexible region of HDAC2 is likely to either undergo a partial collapse or an expansion in the gas phase resulting in low and high charge state distributions. However, our crosslinking experiments were performed in-solution, and the IM-MS spectra for both crosslinked and non-crosslinked samples agree (Fig. 2C). Importantly, the crosslinking data was used as modelling restrains as these data could capture the solution state protein.

### 4.4 Solution-state behaviour of the full-length HDAC2

Previous reports suggest that HDAC2 protein dynamics are regulated by the C-terminal region [1,4]. Because of the flexibility of this region, there is an ensemble of conformations and interaction points between globular and disordered regions that we were able to study using MS. It was observed that following the removal of the C-terminal domain, the activity of the enzyme increased dramatically, while upon crosslinking of the protein, the activity was reduced (Fig. 2D). These data allowed us to hypothesise that the flexible region may interfere with the active site of the protein. In addition to the activity assay results, it was shown that in the mass spectrum of the proteolytically-treated HDAC2, the high charge state species distribution was not observed. This further suggests that the unfolded-like behaviour shown in the native mass spectra is due to the high protonation ability of the intrinsically-disordered region.

**Fig. 5:**
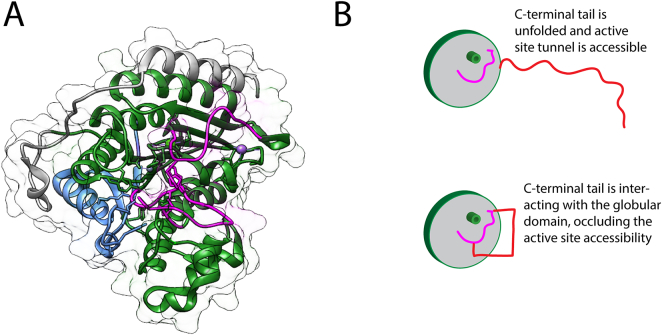
Model of how the C-terminal tail is involved in HDAC2 regulation. (A) Crystal structure of HDAC2 (PDB ID: 3MAX), coloured by regions: blue - oligomerisation, green - deacetylase and grey - regulatory. In magenta are two loops that were identified in crosslinking experiments and are located close to the active site tunnel of the protein. (B) Proposed mode of action of the C-terminal unstructured region (shown in red), which keeps the active site (green cylinder) exposed when away from the protein, but when it is interacting with the globular domain, it occludes the loop region (magenta) and hereby limits the accessibility of the active site. (For interpretation of the references to colour in this figure legend, the reader is referred to the web version of this article.)

The crosslinking experiments made possible to determine residues on both globular and intrinsically-disordered domains of HDAC2 that come in close proximity and are likely to interact and that way regulate the accessibility to the active site. These data were used to build a model capturing the intrinsic flexibility of the flexible domain. This model helps to explain how the landscape populated by the C-terminal tail region regulates the activity of the enzyme (Fig. 4, Fig. 5).

### 4.5 Proposed mode of action of the intrinsically-disordered domain of HDAC2

In top-scoring 10 models only one model on the intrinsically-disordered domain occludes the active site. This suggests that the area near the active site is targeted more commonly (Fig. 4C). Interestingly, there are two loop regions (residues 140–158 and 207–220, Fig. 5A in magenta), that are overlaying with the interaction region between the globular and the intrinsically-disordered domains (Figs. 4C and 5A). The importance of loop regions in the activity of HDAC enzymes was previously highlighted [52,53]. We propose that in the full-length HDAC2 protein, the interrelation between the flexible loops in HDAC2 and HDAC2 C-terminal domain (Fig. 5A) are affecting how the activity of the enzyme is regulated. When the intrinsically-disordered region of the C-terminus is unfolded, the active site of the HDAC2 is more accessible, however the intrinsically-disordered region can come into proximity with the loop regions, which is likely to occlude them and change the accessibility to the active site of the enzyme (Fig. 5B). Dissecting these relationships is key in better understanding how the enzyme activity is being regulated in human bodies, and can also not only help to guide future MMoA design of HDAC2 inhibitors as well as help with dose prediction models.

## 5 Concluding remarks

This work demonstrates use of multiplexed MS data from denaturing MS, Native MS, IM-MS and crosslinking MS to acquire structural insights into a difficult-to characterise chimeric protein. Here we have shown the power of combining these techniques with molecular modelling approaches to understand protein activity and provide insight into structure-targeted drug discovery, which can lead to the discovery and development of more specific inhibitor molecules. Such combined approach would be useful in studying the structure of other proteins that also contain large disordered regions.

## Author contributions

ZS designed and performed the experimental work and analysis (bacterial expression, protein purification, MS and activity assays) under the supervision of KT and DFH. ACS provided construct for HDAC2 C-terminal expression and assisted with protein purification. JEN and RJO expressed full-length HDAC2. JMAB and MT performed molecular modelling, ZS and JMBJ performed crosslinking data analysis. ZS and KT prepared the manuscript with contribution from other authors.

## Competing financial interests

Authors declared no competing financial interests.

## Declaration of Competing Interest

The authors declare that they have no known competing financial interests or personal relationships that could have appeared to influence the work reported in this paper.

The authors declare the following financial interests/personal relationships which may be considered as potential competing interests:

## Appendix A Supplementary data

Supplementary material Image 1
