# Supplementary material for: Structural mass spectrometry decodes domain interaction and dynamics of the full-length Human Histone Deacetylase 2
Source: Biochim Biophys Acta Proteins Proteom. 2022 Mar 1;1870(3):140759. doi: 10.1016/j.bbapap.2022.140759 (PMC8825994; doi:10.1016/j.bbapap.2022.140759)
Supplement: Supplementary file 1 — Supplementary material [file mmc1.pdf]

Structural mass spectrometry decodes domain interaction and dynamics of the full-length Human Histone Deacetylase 2.

Supplementary information

Construct name	aa sequence	aa number	pI	Theoretical MW (SS/SH) in Daltons
Full-length HDAC2	MAYSQGGGKKKVCYYYDGDIGNYYYGQ GHPMKPHRIRMTHNLLLNYGLYRKMEIY RPHKATAEEMTKYHSDEYIKFLRSIRPDN MSEYSKQMQRFNVGEDCPVFDGLFEFC QLSTGGSVAGAVKLNRQQTDMAVNWA GGLHHAKKSEASGFCYVNDIVLAILELLKY HQRVLYIDIDIHHGDGVEEAFYTTDRVM TVSFHKYGEYFPGTGDLRDIGAGKGKYYA VNFPMRDGIDDESYGQIFKPIISKVMEM YQPSAVVLQCGADSLSGDRLGCFNLTVK GHAKCVEVVKTFNLPLLMLGGGGYTIRN VARCWTYETAVALDCEIPNELPYNDYFEY FGPDFKLHISPSNMTNQNTPEYMEKIKQ RLFENLRMLPHAPGVQMQAIPEDAVHE DSGDEDGEDPDKRISIRASDKRIACDEEF SDSEDEGEGGRRNVADHKKGAKKARIEE DKKETEDKKTDVKEEDKSKDNSGEKTDT KGTKSEQLSNPKLEVLFQGPDYKDDDDK LEHHHHHHHH	515	5.66	58700/58710
C-terminal HDAC2 truncate (after TEV protease cleavage)	GPDFKLHISPSNMTNQNTPEYMEKIKQ RLFENLRMLPHAPGVQMQAIPEDAVH EDSGDEDGEDPDKRISIRASDKRIACDE EFSDSEDEGEGGRRNVADHKKGAKKA RIEEDKKETEDKKTDVKEEDKSKDNSGE KTDTKGTKSEQLSNP	150	4.77	16912/16913

Table S1. Sequence information for full-length and C-terminal HDAC2 construct used in this study.

Mass-to-charge	Charge State	mass	replica
1624.41	+5	8117.05	1
1353.86	+6	8117.16	1
1624.40	+5	8117.00	2

1353.82	+6	8116.92	2
1624.59	+5	8117.95	3
1353.96	+6	8117.76	3

Table S2. Mass calculations for the additional peptide resulting from HDAC2 limited proteolysis experiments. Mass is calculated from three different mass spectra replicas acquired on separate days, the average mass is 8,117.31 ± 0.4 Da. The experimentally-calculated mass corresponds closely to the theoretical peptide value between residues 447-515 (8,116.6 Da), where residue 446 is targeted by trypsin.

Sequence	Average Theoretical MW
1-58 aa	7076.18
406-515 aa	12631.56
447-515 aa	8116.65

Table S3. Theoretical mass calculations of possible limited proteolysis-resulting peptides when estimating from sequence coverage (Figure S1). The resulting largest N-terminal peptide sequence is smaller than observed mass, and peptide between residues 447-515 is a close match to the observed theoretical masses (Table S2).

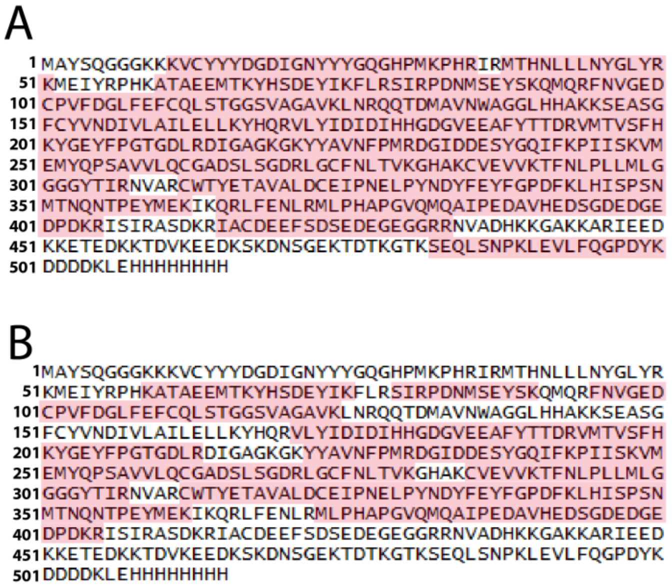

Fig. S1. Sequence coverage highlighted in red of either full or partially-matched peptides of the full-length HDAC2 sample in **(A)**. or the proteolytically-treated HDAC2 in **(B)**. Peptides were identified by LC-MS/MS sequencing.

High CSD	Low CSD

Charge state	CCS value (Å^2)	Charge state	CCS value (Å^2)
+31	7675±138	+14	3854±89
+32	7912±198	+15	3986±98
+33	8174±234	+16	4135±101
+34	8412±251		
+35	8613±258		
+36	8622±254		

Table S4. Experimental CCS values of the full-length HDAC2 for both high and low charge state distribution. Values were extracted from three different mass spectra acquired on three different days. The average values and error were calculated for each charge state and are indicated in the table.

Model	MNXL	CCS TJM
1	-6.10	4284
2	-6.26	4513
3	-6.53	4760
4	-6.56	4497
5	-6.56	4616
6	-6.60	4531
7	-6.62	4354
8	-6.65	4588
9	-6.68	4512
10	-6.70	4421

Table S5. MNXL score values and theoretical CCS values for the top scoring 10 models of HDAC2 C-terminus. Theoretical values were calculated using IMPACT [31].

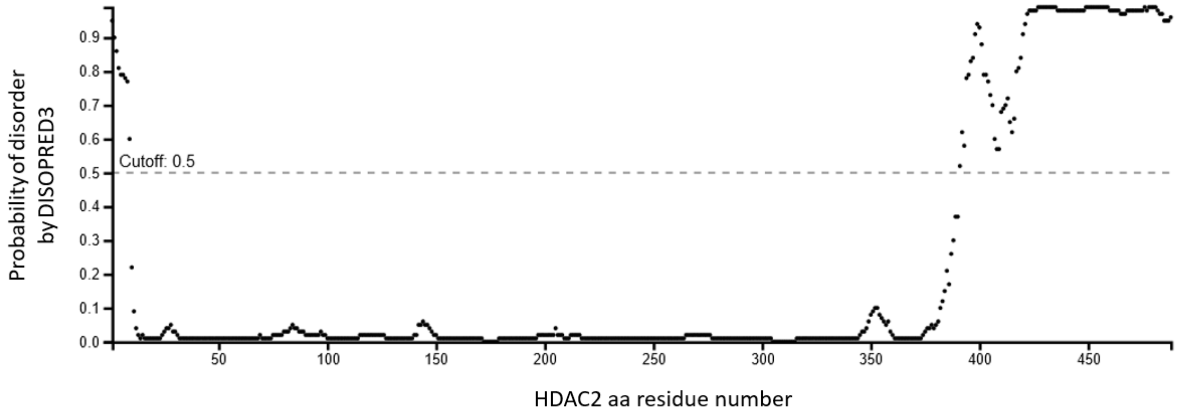

Fig. S2. Disorder prediction for HDAC2 FL sequence calculated using DISOPRED3 [3].
